# 

**Published:** 1956-07

**Authors:** 


### THE MEDICAL JOURNAL OF THE SOUTH-WEST

*Incorporating*
*The Bristol Medico-Chirurgical Journal*

ARMED FORCES

AUG 2 1956

MEDICAL LIBRARY

*July 1956*

VOL. 71 (iii). No. 261 Price 4/-

